# Acceptability and utility of an electronic psychosocial assessment (myAssessment) to increase self-disclosure in youth mental healthcare: a quasi-experimental study

**DOI:** 10.1186/s12888-015-0694-4

**Published:** 2015-12-01

**Authors:** Sally Bradford, Debra Rickwood

**Affiliations:** 1 Faculty of Health, University of Canberra, Building 12, D20, College St, Bruce, ACT 2617 Australia; 2 Faculty of Health, University of Canberra, Building 12, D11, College St, Bruce, ACT 2617 Australia; 3 headspace National Youth Mental Health Foundation, Melbourne, VIC Australia

**Keywords:** Youth, Adolescence, Mental health, Technology, Psychosocial assessment, Self-disclosure

## Abstract

**Background:**

Technology is increasingly being used in youth mental healthcare to support service delivery and improve health outcomes. The current study trialed a new electronic psychosocial application (myAssessment) that aims to provide a holistic assessment of relevant risk and protective factors in youth mental healthcare. The study aimed to determine whether myAssessment was acceptable to all users, and whether it affected: reporting of certain behaviors and ratings of self-disclosure; youth ratings of control, fears of judgmental reactions or time-efficiency; clinician ratings of time-efficiency or their ability to formulate a treatment plan; and the therapeutic alliance.

**Method:**

The application was tested at a youth mental health service using a quasi-experimental two phase Treatment-as-Usual/Intervention design. Three hundred thirty nine youth and 13 clinicians participated across both phases. Reporting of behaviors, self-disclosure, youth control, judgmental reactions, time efficiency, ability to formulate treatment plans, and the therapeutic alliance were compared between groups.

**Results:**

myAssessment was found to be widely accepted by both young people and clinicians. Use of myAssessment resulted in reporting of behaviors that were 2.78 through 10.38 times higher for a variety of substances (use of tobacco, alcohol, cannabis, sedatives, hallucinogens, and opioids), in identifying non-heterosexual sexual orientation, having had sex, an STI check, sex without a condom, having felt pressured to have sex in the past, having self-harmed, and in having put themselves in an unsafe situation. Participants who used the application also reported being less likely to lie on past experiences of being bullied, substance use, and self-harm. Use of the application resulted in improved youth ratings of time efficiency in session. The application was found to have no impact on youth control, judgmental reactions, formulation of treatment plans, or the therapeutic alliance.

**Conclusions:**

Electronic psychosocial assessments can increase rates of self-disclosure and, therefore, provide an earlier and more comprehensive picture of young people’s risks without negatively impacting the therapeutic alliance. Additionally, this type of technology has been shown to be widely accepted by both young people and clinicians and can improve youth beliefs that there is enough time in session to speak about what is most important to them.

## Background

Adolescence and young adulthood are turbulent periods of development— not only because they involve navigating through the normal (but often challenging) social, educational, occupational, and financial transitions to adulthood, but also because a person is at the highest risk of having a mental disorder compared with any other point across the lifespan. With half of all lifetime cases of mental disorders emerging before 14 years, and 75 % emerging by 24 years [1], it is imperative that targeted support is provided to this vulnerable group. Over the last decade one of the major reforms to mental health care has been establishing youth focused services that provide support to young people across the full 12–25 year range to ensure they receive the appropriate care throughout the entire transitional period [2]; this is in contrast to earlier models that utilized separate child and adult services where a young person would be required to navigate a starkly different service model at the age of 18 years [3]. Australia has been at the forefront of this reform [4], with the establishment of youth specific services such as ‘headspace National Youth Mental Health Foundation’ [5–7], ‘ReachOut.com by Inspire Foundation’ [8, 9], and ‘Orygen Youth Health’ [10]. In more recent years, mental health reforms for young people have extended to include a particular focus on how technology can be utilized to improve service delivery and mental health outcomes [11, 12]. In the current study, we further progress in this direction by investigating whether an electronic psychosocial assessment application, ‘myAssessment’, could support initial assessments in face-to-face therapy with young people.

In the provision of mental healthcare, clinicians should be adhering to principles of evidence-based practice in psychology (EBPP) [13]. Specifically, this includes conducting a comprehensive psychological and psychosocial assessment to be used in case formulation and treatment planning, the provision of empirically supported therapeutic interventions, and ongoing monitoring of client progress via outcome data. In line with recommendations from governing agencies, a comprehensive psychological and psychosocial assessment should cover multiple emotional, behavioral, social, and environmental contexts; this includes but is not limited to age, developmental history, ethnicity, housing, education, race, gender, sexual orientation, religious commitments, and socioeconomic status [13–16]. While obtaining this information is an essential first step in therapeutic case formulation and treatment planning, such a comprehensive assessment can take considerable time and may be perceived as invasive and disengaging to the client. Additionally, EBPP also highlights the importance of early establishment and maintenance of the therapeutic relationship to ensure clients continue to engage in therapy and obtain positive therapeutic outcomes [13, 17, 18]. This is particularly important when working with young people as they are highly unlikely to return to services if they were unhappy with the initial experience [19, 20]. Thus, clinicians must delicately balance the necessity of obtaining comprehensive clinical information early in treatment, while fostering a caring and supportive relationship to ensure a young person returns for ongoing support.

This difficult balancing act may be supported by utilizing the benefits of technology. With the ever increasing use of smart phone technology and social networking, the notion that we have a separate online and offline identity is now outdated [21]. As such, mental health reforms are increasingly looking into how technology can be integrated into mental health services to engage young people in the mediums in which they are now so accustomed [11, 22]. In the early stages of face-to-face therapy, utilizing technology in the initial assessment process is likely to be beneficial for a number of reasons. Firstly, young people generally prefer self-administered assessments over those that require verbal face-to-face disclosure, as they provide young people greater control over the disclosure process and time to organize their thoughts [23–25]. There is also greater user satisfaction with questionnaires that utilize technology [26] as computerized assessments are generally more engaging and can incorporate response logic targeted to the user [27, 28]. Finally, a large systematic review has shown that online formats may result in a greater frequency of initial disclosures of personal information [29]. Thus, an online assessment may result in greater reporting of risky behaviors by young people.

In response to these possible benefits, an electronic assessment application ‘myAssessment’ was developed to help obtain information quickly and easily for use in subsequent face-to-face therapy. myAssessment is suitable for young people across the full 12–25 year age range and is based on the widely used semi-structured interview ‘HEADDSSS’ (**H**ome, **E**ducation/Employment, **A**ctivities and Peer relations, **D**rugs and Alcohol, **S**exuality, **S**uicide/Depression, and **S**afety), which assesses young people across multiple domains relevant to their mental health and psychosocial functioning [30, 31]. headspace National Youth Mental Health Foundation has adapted the interview to cover the domains of Home and Environment, Education and Employment, Activities, Alcohol and Other Drugs, Relationships and Sexuality, Conduct Difficulties and Risk-taking, Anxiety, Eating, Depression and Suicide, and Psychosis and Mania [32, 33]. myAssessment follows the headspace Psychosocial Assessment for Young People [33] with an added inclusion of Physical Health, and specific questions about past bullying experiences in school and the workplace. Similar language is used in myAssessment as in the screening and probing questions within the headspace interview guide [33]. The number of total items in myAssessment varies between 62 and 93 based on individual responses, and takes approximately 10–15 min to complete. On completion, myAssessment provides an automated ‘Clinician Summary’ that highlights personal strengths and areas of risk in each domain. The information provided in the Clinician Summary is subsequently used as a foundation in the first face-to-face appointment where clinicians can verbally probe to obtain a greater depth of understanding around any identified areas of risk or concern for the young person.

myAssessment was developed using human-centered design principles [34] which involved extensive research with stakeholders in order to determine functionality requirements and attitudes and beliefs around such an application [25, 35, 36]. In addition to the previously identified benefit of potentially obtaining higher rates of initial disclosure [29], this preparatory research highlighted other possible benefits and concerns. Both young people and clinicians felt that by using the application prior to the first appointment, young people would be given increased control in the treatment process as they could organize their thoughts and indicate what it was that they most wanted to speak about via a ‘flagging’ function [25, 35, 36]. Both groups also felt that the application would offer greater time efficiency as they could focus more on the important issues in session and spend less time on gathering less necessary information [25, 35]. Separately, young people felt that using the application would decrease their fears that their responses might be negatively judged by the clinician [25], and clinicians felt the extra information obtained early in the clinical process could help them formulate a treatment plan [35]. Nevertheless, while clinicians reported some possible benefits, most were primarily concerned that the application would negatively impact on therapy by not providing an accurate representation of the young person and by interfering with the therapeutic alliance [35].

### Current study

The current study trialed myAssessment in a youth specific mental health service to determine whether the application: 1) was acceptable to both young people and clinicians, and whether it was able to provide an accurate representation of each young person; 2) increased reporting of behaviors and levels of self-disclosure of personal or risky behaviors; 3) affected youth feelings of control in-session, fears of judgmental reactions, and time efficiency; 4) affected clinician ratings of time efficiency, or their ability to formulate a treatment plan; and 5) affected the therapeutic alliance as determined by both young people and clinicians. For the second aim, the use of myAssessment was hypothesized to result in higher reporting of behaviors disclosed, increased ratings of self-disclosure, and less lying, particularly in the domains considered embarrassing or highly personal. Secondly, it was hypothesized that young people who used the application would provide increased ratings of control, reduced fears of judgmental reactions, and improved ratings of time efficiency. In response to the fourth aim, it was anticipated that myAssessment would result in improved clinician ratings of time efficiency in session, and in an improved ability to formulate a clear treatment plan. Finally, evaluating the effect upon the therapeutic alliance as rated by both young people and clinicians was exploratory in nature and no directional hypothesis was made.

## Method

### Participants

#### Youth participants

Participants were 339 young people attending their first appointment at ‘headspace Canberra’ in the Australian Capital Territory, from April to December 2014. All young people attending headspace for their first appointment during this time (*N* = 386) were invited to participate. Forty-seven declined, resulting in a full response rate of 87 %. Demographic information is presented in Table 1.

**Table 1:** Description of the youth sample

		Control (*n* = 188)	Intervention (*n* = 151)	Total (*N* = 339)	Group comparison
Gender					Fisher exact test, *p* = .002
	Female	124 (66 %)	95 (62.9 %)	219 (64.6 %)	
	Male	59 (31.4 %)	39 (25.8 %)	98 (28.9 %)	
	Trans*/intersex	2 (1.1 %)	1 (.7 %)	3 (.9 %)	
	Other/not stated	3 (1.6 %)	16 (9.6 %)	19 (5.6 %)	Std. Residual > 1.96
Age, y					
	Mean (SD)	17.72 (3.5)	17.2 (3.45)	17.49 (3.49)	
	Range	12—25	12—25	12—25	
Sexual preference					
	Heterosexual/straight	135 (71.8 %)	108 (71.5 %)	243 (71.7 %)	
	Lesbian/gay	7 (3.8 %)	1 (.7 %)	8 (2.4 %)	
	Bisexual	15 (8 %)	13 (8.6 %)	28 (8.3 %)	
	Questioning	5 (2.7 %)	9 (6 %)	14 (4.1 %)	
	Other/choose not to answer	26 (13.9 %)	20 (13.2 %)	46 (13.6 %)	
Aboriginal and Torres Strait Islander (ATSI) Status					*x* ^2^ (1, *N* = 339) = 1.00, *p* = .318
	ATSI	4 (2.1 %)	6 (4 %)	10 (3 %)	
	Neither/not stated	184 (97.9 %)	145 (96 %)	329 (97 %)	
Country of birth					
	Australia	159 (84.6 %)	128 (84.8 %)	287 (84 %)	
	New Zealand	2 (1.1 %)	1 (.7 %)	3 (.9 %)	
	South Africa	1 (.5 %)	-	1 (.3 %)	
	England	-	2 (1.3 %)	2 (.6 %)	
	Other/not stated	26 (13.8 %)	20 (7.9 %)	46 (13.5 %)	
Language spoken at home					Fisher exact test, *p* = .518
	English	158 (84 %)	128 (84.8 %)	286 (84.4 %)	
	Hindi	-	2 (1.4 %)	2 (.6 %)	
	Spanish	2 (1.1 %)	1 (.7 %)	3 (.9 %)	
	Other/not stated	28 (14.6 %)	20 (13.2 %)	48 (27.6 %)	

Note. Trans* is an inclusive term encompassing the following: Transgender, transsexual, genderqueer, non-binary, genderfluid, genderfuck, intersex, third gender, transvestite, cross-dresser, bi-gender, trans man, trans women, agender

*Note*. Trans^*^is an inclusive term encompassing the following: Transgender, transsexual, genderqueer, non-binary, genderfluid, genderfuck, intersex, third gender, transvestite, cross-dresser, bi-gender, trans man, trans women, agender

#### Clinicians

Thirteen clinicians participated in the study; six females and seven males. Six of the clinicians worked in the center as a private practitioner (46.2 %) and seven were employed as staff on salary (53.8 %). Seven of the clinicians were employed in a psychologist role (53.8 %), and six were employed as a Youth Mental Health and Community Worker (46.2 %). Educational backgrounds included psychology (69.23 %), social work (15.39 %), and counselling (15.39 %).

### Centre

‘headspace Canberra’ is part of headspace National Youth Mental Health Foundation, which is a youth specific mental health service with multiple centers across Australia. The organization offers a range of health services including general health, mental health and counseling, education and employment support, and alcohol and other drug services to young people aged 12–25 years [5, 6]. ‘headspace Canberra’ provides services to a diverse range of young people across the entire Canberra, ACT and Queanbeyan, NSW region, Australia.

### Design and procedure

Research ethics approval was first obtained from the University of Canberra Committee for Ethics in Human Research (UC CEHR) (Approval no. 14–21). All participants signed a consent form, and participants aged 14 years or younger also required signed parental consent. The requirement of parental consent for participants aged 15–16 years was waived by the UC CEHR as headspace Canberra does not require consent for this age group to attend their service. Requesting consent from these participants would have affected their right to receive confidential mental health support or unnecessarily excluded them from the study. Each participant received a $5 gift voucher for participating. A quasi-experimental two phase design was utilized with a Treatment-As-Usual (TAU) phase running from April 2014 through to July 2014, and an Intervention phase, running from July 2014 through December 2014.

In the TAU phase, youth participants attended their initial appointment where they were expected to undergo a verbal psychosocial assessment. The headspace Psychosocial Assessment for Young People [33] is a semi-structured interview delivered to young people attending initial appointments at headspace centers. It is an expected component of the headspace model of service delivery and staff are provided training in the use of the assessment. The interview covers the following domains: Home and Environment, Education and Employment, Activities, Alcohol and Other Drugs, Relationships and Sexuality, Conduct Difficulties and Risk-taking, Anxiety, Eating, Depression and Suicide, and Psychosis and Mania. For each domain the guide provides initial screening questions and additional probing questions. For the domains of ‘Alcohol and Other Drugs’ the guide suggests the additional use of The Alcohol, Smoking and Substance Involvement Screening Test (ASSIST) [37]. ASSIST asks which specific substances a young person has used in their life, followed by the frequency of use of that substance in the previous three months. The domain of Relationships and Sexuality includes specific probing questions around past sexual experiences and sexual health behaviors such as “*Have you had sex in the last 12 months?*” and “*Have you been tested for sexually transmitted infections in the last 12 months*?” The Conduct Difficulties and Risk Taking domain also includes specific questions about self-harm and unsafe-behaviors such as “*Have you deliberately harmed or injured yourself – like cutting, burning or scratching yourself – when not feeling suicidal*?”, “*Have you put yourself in unsafe situations (e.g. unsafe sex, risky driving)?*” “*When did it start? How Often?*” At the conclusion of this initial appointment, both young people and clinicians completed an anonymous post-session questionnaire. The clinicians’ questionnaire included questions on rates and frequencies of behaviors (as verbally reported in the headspace Psychosocial Assessment for Young People) as well as the post-session measures of Time Efficiency, Formulation of a Treatment Plan and the Therapeutic Alliance. The youth questionnaire included demographic questions and measures of Self-Disclosure, Youth Control, Judgmental Reactions, Time Efficiency, and Therapeutic Alliance.

In the Intervention phase, participants responded to the same questions as in the headspace Psychosocial Assessment for Young People, but via myAssessment rather than verbally. The Clinician Summary was then automatically provided to the clinician for use in the subsequent face to face session. At the conclusion of this session, clinicians completed a questionnaire asking them to rate whether myAssessment provided them with an accurate picture of that young person and their psychological functioning, and included the same post-session measures as the TAU phase. Youth participants also completed the same anonymous post-session questionnaire as those in the TAU phase, but with additional questions on myAssessment acceptability. Data on rates and frequencies of behaviors (as electronically reported) were manually extracted from each participant’s completed myAssessment form.

Of the total 339 participants, 274 provided data on reporting of behaviors and completed the post-session questionnaire, 38 only provided reports of behaviors, and 27 only completed the post-session questionnaire. Post-study, each clinician was also asked to complete a final questionnaire about their overall experience of using myAssessment.

### Measures

#### Youth acceptability

Youth participants in the Intervention phase rated the following six questions on a scale from 1 (*Not at All)* through 5 (*Very Much*): ‘Was myAssessment easy to use? How confident are you that myAssessment was able to provide an accurate picture of yourself to the therapist/counsellor? How comfortable were you disclosing personal information about yourself through myAssessment? Were the questions easy to understand? Did any of the questions in myAssessment cause you to become upset? And, Were you comfortable completing myAssessment in the waiting room?’ Questions were developed based on qualitative comments provided during the preparatory research phase [25]. Responses for each question were analyzed separately.

#### Clinician acceptability

Clinicians rated the following five questions on a scale from 1 (*Not at All*) through 5 (*Very Much*): ‘Was the clinician summary easy to follow? Overall, how confident are you that myAssessment clinician summary will provide you with an accurate picture of a young person’s mental health problems? Overall, how confident are you that myAssessment clinician summary will provide you with an accurate picture of a young person’s psychosocial functioning? How useful is myAssessment clinician summary in helping you obtain a full mental health and psychosocial history? If you had the choice, would you continue using myAssessment in your clinical work?’ Each question was analyzed individually.

#### Reporting of behaviors

Reporting of behaviors was compared across the TAU and Intervention groups. In the TAU phase, clinicians provided information on each young person they treated based on what the young person had verbally reported during the initial headspace Psychosocial Interview, for each of the following domains: sexuality status, substance use (tobacco, alcohol, cannabis, cocaine, amphetamine, inhalants, sedatives, hallucinogens, opioids), sexual health, self-harm, and unsafe situations. The questions used the same language as the screening and probing questions in the headspace Psychosocial Assessment for Young People [33]. For example, “Has the young person deliberately harmed or injured themselves (like cutting, burning, or scratching them self – when not feeling suicidal” and “Has the young person ever had sex? (This includes oral, anal or vaginal)” The initial response options were ‘*Yes,*’ leading to further questions about when the most recent behavior had occurred from 1 (*Past Month*) through 4 (*Over 12 Months Ago*), or *‘No/Did not Disclose’.* The substance use questions followed the suggested ASSIST format [37] by first asking, “In their life, what substances has the young person used?” Clinicians then selected all that apply from the following: Tobacco, Alcoholic Beverages, Cannabis, Cocaine (coke, crack, etc.), Amphetamines (Speed, diet pills, ecstasy), Inhalants (nitrous, glue, petrol, paint thinner), Sedatives (Valium, Serepax, Rohypnol, etc.), Hallucinogens (LSD, acid, mushrooms, PCP, SpecialK), Opioids (Heroin, morphine, methadone, codeine). For any selected substances clinicians were asked to rate usage in the previous three months from 1 (*Never*) through 5 (*Daily or Almost Daily*).

In the Intervention phase, young people answered questions relating to each of the above domains in the same language and format as clinicians, but via myAssessment prior to their first session. For example, questions included, “Have you deliberately harmed or injured yourself (like cutting, burning, or scratching yourself) – when not feeling suicidal” and “Have you ever had sex? (This includes oral, anal or vaginal)”. The substance use questions also followed the ASSIST format by asking, “In your life, which of the following substances have you ever used?” with young people selecting all that apply for the substances stated above. Selected substances were then rated on use in the previous three months.

Each behavior or substance was compared independently between groups.

**Self-Disclosure** was assessed using a measure adapted from the Jourard Sixty-Item Self-Disclosure Questionnaire [38] and a measure developed by Mallen et al. [39]. The rating scale was adapted by increasing the scale from four to six points, with participants rating themselves from 0 (*I have lied or misrepresented myself*), 1 (*No Disclosure = I have told the counsellor/therapist nothing about this respect of me*) through 5 (*Extreme Disclosure = I have talked in full and complete detail about this item. They know me fully in this respect and could describe me accurately)*. Participants provided a rating for each of the 10 items relating to different myAssessment domains: Physical Health, Home Life, School, Bullying experiences, Alcohol and Other Drug use, Relationships, Sexual Orientation, Self-harming Behaviors, Unsafe Situations, Mood, and Worry. Scores were assessed separately for each domain and were also summed to obtain a ‘Total Self-Disclosure’ score with higher scores indicating greater overall self-disclosure.

#### Youth control

Youth feelings of control within session were measured using an adapted version of the Cohesiveness Concerns subscale from the Thoughts About Psychotherapy Survey (TAPS) [40]. The scale was adapted by changing the language from future to past tense, and an additional item, “The questions the therapist/counsellor asked me took me by surprise”, was added based on prior research [25]. Each of the four items was rated on a five-point scale from 1 (*Not at All*) through 5 (V*ery Much*). All items were then reverse scored and averaged to obtain a total score with higher scores indicating greater feelings of control within session.

#### Judgmental reactions

Fears of Judgmental Reactions were measured using an adapted version of the ‘Image Concerns’ subscale from the Thoughts About Psychotherapy Survey (TAPS) [40]. The adaptation changed the language from future to past tense, and an additional item, “That the counsellor/therapist would judge me”, was added. Each of the six items was rated on a five-point scale from 1 (*Not at All Concerned*) through 5 (*Very Concerned*), and averaged to obtain a total score, with higher scores indicated greater fears of judgement from the clinician.

#### Time efficiency

Youth ratings of time efficiency were measured from the single item, “I felt that there was enough time in session to talk about what was most important to me today”. Clinician beliefs were rated using the single item, “I felt that there was enough time in session to cover everything I wanted to cover with the young person”. Both were rated on a five-point scale from 1 (*Not at All*) to 5 (*Very Much*).

#### Formulation of treatment plan

Formulation of a treatment plan was rated by clinicians with the single item, “I have a clear idea about the treatment approach I will take with this client”, rated on a five-point scale from 1 (*Not at All*) through 5 (*Very Much*).

#### Therapeutic alliance

Youth ratings of the therapeutic alliance were measured using the Therapeutic Alliance Quality Scale-Youth (TAQS-Youth) [41]. Responses on the five items were scored on a five-point scale from 1 (*Not at All*) through 5 *(Totally)*. Scores were averaged, with higher scores indicating greater Therapeutic Alliance. Scores under 3.8 are considered ‘low’, scores of between 3.8 and 4.8 are considered ‘medium’, and scores over 4.8 are considered ‘high’ [41].

Clinician views of the Therapeutic Alliance were rated using the single item, “In this session, how would you describe your relationship with this young person?” from the Therapeutic Alliance Quality Rating scale (TAQR) [41]. Responses were scored on a five-point scale from 1 (*Not at All*) through 5 *(Totally)* with higher scores indicating greater Therapeutic Alliance.

### Statistical analysis

Data were analyzed using PASW Statistics 21 [42] with alpha set at .05. All data were first carefully screened with less than 5 % missing. The TAU and Intervention groups were compared on a range of demographic variables to ensure they were comparable. The only noted difference was in Gender, where significantly more people in the Intervention group identified as Other/Not Stated (See Table 1). The psychometric properties of each scaled variable with more than one item are presented in Table 2. All scales attained adequate internal consistency according to Cronbach’s alpha. Skewness scores showed a major deviation from normality for the scale Youth Control; a ‘reflect and inverse’ transformation for extremely negatively skewed data did not adequately improve the results, thus this was variable was tested using the non-parametric Mann–Whitney *U* analysis. Prior to each other analysis, all other assumptions were tested and met.

**Table 2:** Psychometric properties of the study variables

					Range		
Variable	No. of items	*M*	*SD*	*α*	Potential	Actual	Skew
Total self-disclosure	10	31.91	11.08	.90	0–50	0–50	−.53
Youth control	4	4.65	.62	.81	1–5	1.00–5.00	−2.77
Judgmental reactions	6	2.15	.88	.79	1–5	1.00–5.00	.97
TAQS-Youth	5	4.14	.62	.78	1–5	2.00–5.00	−.51

In comparing verbal (TAU) vs electronic (Intervention) reporting of sexual preference, the categories of Lesbian, Gay, Bisexual and Questioning were combined to create a single ‘Non-heterosexual’ category as each cell frequency was too low for individual comparisons. Groups were therefore compared between the options of ‘Heterosexual’ and ‘Non-heterosexual’. In order to compare groups on the likelihood of lying in each domain, scores on the measure of self-disclosure were transformed into a dichotomous variable. Scores of ‘0’ remained as a single category of ‘I lied’, and any score of 1–5 were combined into the single category ‘I disclosed something about myself’.

Pearson’s Chi-square Test of Contingencies were used to compare rates of disclosure and the likelihood of lying on each domain, between groups. In any analysis where 50 % of cells had a minimum frequency of less than 5, the Fisher’s exact test is reported. In cases where the analysis was significant and larger than a 2 X 2 analysis, separate Post-Hoc 2 X 2 Chi-square tests were subsequently conducted. Mann–Whitney *U* tests were used to compare the reported amounts of substance use and most recent behaviors between groups, and to compare groups on the non-parametric variable ‘Youth Control’. The mean rank was used in analyses where distributions were not similar. In variables where *n* was fewer than 20 in either group, the exact sampling distribution for *U* is reported. Independent-samples *t*-tests were used to compare groups on reported levels of Self-Disclosure, Judgmental Reactions, Time Efficiency, Formulation of a Treatment Plan, and Therapeutic Alliance. In cases where homogeneity of variance was violated the ‘Equal variance not assumed’ statistic is reported.

## Results

### Acceptability of myAssessment

#### Youth

Overall, myAssessment was widely accepted by youth. Ninety-two percent rated the application as being either ‘Quite’ or ‘Very’ easy to use, and 74.4 % were ‘Quite’ or ‘Very’ confident that it provided an accurate picture of themselves. The majority felt either ‘Quite’ or ‘Very’ comfortable using the application to disclose personal information and were happy to do so in the waiting room (76.7 and 73.6 %, respectively). Eighty-nine percent felt that the questions were ‘Quite’ or ‘Very’ easy to understand, and only 7 % reported being ‘Quite’ or ‘Very’ upset by any of the questions.

#### Clinician

In almost two-thirds of individual youth sessions (61.7 %), and at a rating of 72.7 % overall, clinicians felt that the myAssessment Clinician Summary provided either a ‘Quite’ or ‘Very’ accurate picture of their client’s mental health. In over half (51.1 %) of individual sessions, and 72.7 % overall, clinicians also believed that the clinician summary provided either a ‘Quite’ or ‘Very’ accurate picture of their client’s psychosocial functioning. Nearly 91 % stated that the Clinician Summary was either ‘Quite’ or ‘Very’ easy to follow, and 72.7 % believed it was either ‘Quite’ or ‘Very’ useful in helping obtain a full mental health and psychosocial history. Ninety-one percent also stated they would be either ‘Quite’ or ‘Very’ likely to continue using myAssessment in their clinical work if the choice was theirs.

### Reporting of behaviors, self-disclosure, and likelihood of lying

The results of the Chi-square analyses are presented in Table 3. This shows that the Intervention group was significantly more likely to report behaviors for the questions of drank or smoked, used tobacco, alcohol, cannabis, sedatives, hallucinogens, and opioids, non-heterosexual sexual orientation, having had sex, an STI check, sex without a condom, whether they had been pressured to have sex, self-harmed, and whether they had put themselves in an unsafe situation. The effect sizes were small to medium, and ranged from .15 to .38. Odds ratios revealed that participants in the Intervention phase were between 2.78 times through 10.38 times more likely to positively report engaging in these behaviors than participants in the TAU phase, with the strongest effects seen in the reporting of sedative and opioid use. Post-Hoc 2 X 2 analyses were conducted for the questions related to self-harm and unsafe situations. The association between group and reporting of self-harm was moderate and statistically significant when comparing the responses of ‘No’ and ‘Yes’, *x*^2^ (1, *N* = 273) = 22.65, *p* < .001, Cramer’s *V* = .29, and ‘No’ vs ‘Thought about it but did not act’, *x*^2^ (1, *N* = 184) = 15.27, *p* < .001, Cramer’s *V* = .29. Odds ratios revealed that participants in the Intervention group were 3.32 times more likely to report that they had self-harmed and 4.32 times more likely to report thinking about self-harming. The association between group and reporting of unsafe situations was small to moderate and statistically significant when comparing the responses of ‘No’ and ‘Yes’, *x*^2^ (1, *N* = 166) = 8.75, *p* = .003, Cramer’s *V* = .18, and ‘No’ vs ‘Thought about it but did not act’, *x*^2^ (1, *N* = 243) = 13.37, *p* < .001, Cramer’s *V* = .24. Odds ratios revealed that participants in the intervention phase were 2.32 times more likely to report that they had been in an unsafe situation, and 5.32 times more likely to report thinking about engaging in an unsafe situation.

**Table 3:** Proportion of participants reporting substance use and other behaviors

	Response of ‘Yes’ (%)						
	Control	Intervention	Pearson Chi-Square (*x* ^2^)	df (*N)*	*p*	Cramer’s V	Odds ratio
Drink or smoke	35.1	60	19.22	1 (311)	<.001^*^	.25	2.78
Tobacco	12.3	39.6	30.81	1 (310)	<.001^*^	.32	4.68
Alcohol	29.2	54	19.47	1 (310)	<.001^*^	.25	2.84
Cannabis	14	39.6	26.32	1 (310)	<.001^*^	.29	4.01
Cocaine	2.9	5	.92	1 (310)	.338	.05	-
Amphetamines	6.4	12.2	3.14	1 (310)	.077	.10	-
Inhalants	1.2	.7	Fisher’s exact	- (310)	1	.02	-
Sedatives	.6	5.8	Fisher’s exact	- (310)	.012^*^	.15	10.38
Hallucinogens	1.8	7.2	5.65	1 (310)	.017^*^	.15	4.34
Opioids	.6	5.8	Fisher’s exact	- (310)	.012^*^	.15	10.38
Non-heterosexual orientation	7.8	21.7	10.81	1(279)	.001^*^	.20	3.28
Had sex	19.9	47.1	25.74	1 (307)	<.001^*^	.29	3.58
STI check	.88	42.18	11.64	1 (98)	.001^*^	.35	7.54
Sex without condom	35.29	73.85	13.90	1 (99)	<.001^*^	.38	5.17
Pressured to have sex	17.64	36.92	4.50	1 (99)	.034^*^	.21	2.91
Self-harmed	31	52.2	28.44	2 (309)	<.001^*^	.30	-
Unsafe situations	15.8	27.2	19.97	2 (307)	<.001^*^	.25	-

^*^ *Significant at p <* .05

Significant differences were found when comparing past levels of use between groups for the substances of Tobacco, Cannabis, Cocaine, and Amphetamine use (see Table 4). In each instance the mean rank was smaller in the Intervention group, indicating that using the application resulted in increased reporting of less common substance use behaviors.

**Table 4:** Reported frequency of engagement in substance use and other behaviours over previous three months

	Median frequency label					
	Control	Intervention	*N*	*U*	*z*	*p*
Tobacco use	Daily	Monthly	71	225	−3.19	.001^*^
Alcohol use	Monthly	Once or twice	120	1341	−1.81	.071
Cannabis use	Weekly	Monthly	75	341	−2.34	.016^*^
Cocaine use	Monthly	Never	12	3.5	−2.39	.018^*a^
Amphetamines use	Once or twice	Never	29	38.5	−2.90	.005^*a^
Inhalants use	Once or twice	Never	2	0	−1	1.00^a^
Sedatives use	Once or twice	Never	9	2.5	−.64	.667^a^
Hallucinogens use	Once or twice	Once or twice	13	10.5	−.88	.469^a^
Opioids use	Weekly	Once or twice	9	1.5	−1.02	.444^a^
Self-harmed	Never	Once or twice	119	1755	.37	.711
Unsafe situations	Once or twice	Once or twice	61	416	−.60	.549

^*^ *Significant at p <* .05; ^a^Fisher’s Exact

Mean reported levels of Self-Disclosure for each myAssessment domain and total Self-Disclosure scores are presented in Table 5. There was greater self-disclosure for the domains of AoD, sexual orientation, and self-harm, with small effect sizes. Additionally, for each of these domains, participants in the Intervention group were significantly less likely to have actively lied (see Table 6). While the Intervention group did not necessarily believe they disclosed more about past bullying experience, they rated themselves as being less likely to actively lie on this domain. Odds ratios revealed that the TAU group was between. Thirty-four times through .Forty-four times more likely to have reported lying on these domains than the Intervention group. The domain of physical health was also approaching significance, with more lying in the TAU group.

**Table 5:** Levels of self-disclosure between groups

	M (SD)						
	Control	Intervention	*df*	*t*	*p*	*d*	95 % CI
Physical health	2.86 (1.54)	2.91 (1.47)	299	−.28	.777	−.03	[−.39, .30]
Home life	3.41 (1.24)	3.51 (1.25)	299	−.69	.491	−.08	[−.38, .19]
School	3.35 (1.44)	3.47 (1.49)	299	−.74	.461	−.08	[−.46, .21]
Bullied	2.58 (1.78)	2.96(1.70)	299	−1.90	.058	−.22	[−.78, .01]
AoD	3.04 (1.91)	3.47 (1.77)	299	−2.01	.046^*^	−.23	[−.85, −.01]
Relationships	3.49 (1.19)	3.41 (1.32)	299	.52	.601	.06	[−.21, .36]
Sexual orientation	2.25 (1.87)	2.77 (1.77)	299	−2.44	.015^*^	−.28	[−.94, −.1]
Self-harm	2.86 (1.86)	3.33 (1.59)	295.94	−2.35	.019^*^	−.27	[−.86, −.08]
Mood	3.60 (1.19)	3.73 (1.3)	299	−.92	.358	−.11	[−.48, .15]
Worry	3.51 (1.20)	3.61 (1.27)	299	.73	.466	−.08	[.39, .18]
Total self-disclosure	30.94 (10.66)	33.18 (11.51)	299	−1.75	.082	−.20	[−4.77, .29]

^*^ *Significant at p <* .05

**Table 6:** Rates of lying in each domain between groups

	% Lied						
	Control	Intervention	Pearson Chi-Square (*x* ^2^)	df (*N)*	*p*	Cramer’s V	Odds ratio
Physical health	11.8	5.3	3.74	1 (301)	.053	.11	-
Home life	4.7	3.8	.14	1 (301)	.707	.02	-
School	8.2	6.1	.50	1 (301)	.482	.04	-
Bullied	17.6	6.9	7.62	1 (301)	.006^*^	.16	.34
AoD	16.5	7.6	5.24	1 (301)	.022^*^	.13	.42
Relationships	2.4	4.6	Fisher’s exact	- (301)	.077	.34	-
Sexual orientation	21.2	8.4	9.17	1 (301)	.002^*^	.18	.34
Self-harm	15.9	7.6	4.67	1 (301)	.031^*^	.16	.44
Mood	2.9	4.6	Fisher’s exact	- (301)	.542	.04	-
Worry	2.9	4.6	Fisher’s exact	- (301)	.542	.04	-

^*^ *Significant at p <* .05

### Youth ratings of control, judgmental reactions, and time efficiency

Ratings of Youth Control was not significantly different between the TAU group (*Mdn* = 5) and the Intervention group (*Mdn* = 4.75), *U* = 9812.5, *z* = −1.75, *p* = .08. However, small to moderate significant effects were found for the youth rating of Time Efficiency, with the Intervention group believing there was significantly more time in session to talk about what was most important to them (see Table 7). No differences were noted between groups on the rating of Judgmental Reactions.

**Table 7:** Ratings of judgmental reactions, time efficiency, formulation of treatment plans and therapeutic alliance between groups

	Control		Intervention						
	*n*	M (SD)	*n*	M (SD)	df	t	*p*	d	95 % CI
Judgmental reactions	169	2.15 (1.02)	131	2.16 (.64)	286.28	−.15	.882	−.01	[−.20, .18]
Time efficiency (youth)	169	4.15 (.85)	131	4.52 (.85)	298	−3.68	<.001^*^	−.44	[−.56, −.17]
Time efficiency (clinician)	171	3.82 (.94)	114	3.68 (1.00)	283	1.16	.249	.15	[−.10, .36]
Formulation of a treatment plan	171	3.97 (.83)	114	3.91 (.98)	212.13	.55	.596	.07	[−.16, .28]
TAQS-Youth	169	4.09 .(63)	131	4.20 (.61)	298	−1.54	.126	−.18	[−.25, .03]
TAQR	171	3.74 (.81)	114	3.80 (.80)	283	−.63	.529	−.07	[−.25, .13]

^*^ *Significant at p <* .05

### Clinician ratings of time efficiency and formulation of a treatment plan

Results were non-significant for clinician ratings of Time Efficiency and Formulation of a Treatment Plan (see Table 7).

### Ratings of therapeutic alliance

Ratings of the Therapeutic Alliance as rated by Clinicians or Young people did not vary between groups (see Table 7).

## Discussion

This study aimed to determine whether myAssessment was acceptable to both young people and clinicians, whether it affected reported levels of behaviors, self-disclosure, time efficiency or experience of the first therapy session. Overall, myAssessment was found to be widely accepted by both young people and clinicians, to have significantly increased reporting of behaviors and self-disclosure in a range of domains, and improved youth ratings of time efficiency. The application was found to have no impact on youth control, judgmental reactions, formulation of treatment plans, or the therapeutic alliance.

Over three-quarters of all young people who used myAssessment stated that they believed it was easy to use, able to provide an accurate picture of themselves, easy to understand, and that they were comfortable using the application to disclose personal information, and to do so in the waiting room. Similarly, just under three-quarters of clinicians believed that overall, the application was useful in providing an accurate picture of young people’s current mental health and psychosocial functioning and in helping them obtain a full mental health and psychosocial history. Further, 91 % stated that the Clinician Summary was easy to follow, and would continue using the application in their clinical work if they were provided the choice to do so. These results are in contrast to previously identified clinician attitudes [35] and supports the notion that while not appropriate for all young people, the vast majority will accept electronically supported psychosocial assessments and find them useful.

There was a small proportion (seven percent) of young people who stated they were upset by some of the items, but it is unclear which specific items this related to, or whether they were generally disconcerted by using the full myAssessment application. Young people in the TAU phase were not asked whether any assessment questions were upsetting, and it is possible that it is the nature of some questions that are upsetting (i.e., self-harm) rather than the form of delivery.

In a systematic review of online and offline self-disclosure rates, Nguyen et al. [29] found that the initial self-reporting of behaviors was higher in an online format, and that this was likely to be most pronounced for topics that are embarrassing or highly personal. Consequently, it was hypothesized that using myAssessment would result in increased reporting of behaviors, and increased ratings of self-disclosure and less lying, particularly in the domains considered embarrassing or highly personal. This hypothesis, and in-turn the previous findings by Nguyen et al. [29] were largely supported. There was a higher percentage of young people reporting engagement in the behaviors of drinking or smoking (use of tobacco, alcohol, cannabis, sedatives, hallucinogens, and opioids), that their sexual orientation was non-heterosexual, that they had had sex, an STI check, sex without a condom, had felt pressured to have sex in the past, had self-harmed, and that they had put themselves in an unsafe situation, by those who used myAssessment as opposed to those who were required to report only verbally. In some instances, young people using myAssessment were over ten times more likely to report information around these topics. Additionally, when comparing rates of previous use of substances, using myAssessment was able to pick up less frequent use of Tobacco, Cannabis, Cocaine, and Amphetamines. Young people who used myAssessment also rated themselves as having higher levels of self-disclosure and having lied significantly less, on topics of alcohol and drug use, sexual orientation, and self-harm, than those who had only the option of verbal disclosure. Use of myAssessment also resulted in significantly less active lying on the domain of bullying. With no significant differences being noted on the topics of physical health, home life, school, relationships, mood, and worry, it does appear that the effects of electronic assessment will be greatest in the areas considered most personal or embarrassing.

It is important to note that while all new clients were expected to undergo the verbal headspace Psychosocial Assessment for Young People and should have been asked questions in the same format as those asked in myAssessment, it is unclear how closely clinicians adhere to this guide in practice. Based on previous research it seems likely that some clinicians may not have felt comfortable enough to have actually asked the most personal questions in session [43, 44]. Consequently, it is not definitively clear whether reporting of behaviors were higher because young people were actually asked the question in a more systematic approach, or because they felt more comfortable providing an honest answer. Although, given that young people also rated themselves on the measure of self-disclosure as *lying significantly less* on the domains of alcohol and drug use, sexual orientation, self-harm, and bullying, it appears that feeling more comfortable in the online format is likely to partly account for the higher rates of reported behaviors.

The capability of myAssessment in supporting the process of reporting and self-disclosure may also increase the likelihood of other young people seeking initial support. Concern in one’s ability to self-disclose, and in their ability to self-disclose *verbally*, has been identified as a barrier to seeking help [20, 45]. Incorporating this type of technology more widely across mental health services will therefore not only likely result in greater reporting by those already seeking help, but may encourage other young people to seek help who are currently too concerned with their ability (or lack there-of) to self-disclose.

It was also hypothesized that young people who used myAssessment would rate themselves as having more control in session, have less fears of judgmental reactions and believe that there was greater time efficiency. This hypothesis was partly supported with there being significantly improved rates of time efficiency, but, in contrast to previous youth and clinician beliefs [25, 35, 36] no changes were noted in fears of judgmental reactions or youth control. Embarrassment, stigma, and fears of what the clinician will think of them have regularly been cited as barriers to service utilization [46, 47] so it is unusual that no differences were noted between groups despite there being significantly greater disclosure. However, it is necessary to note that fears about judgmental reactions from others were already low for those in the TAU group. The headspace services are specifically focused on being youth-friendly and non-judgmental to reduce the barriers to youth mental health service access. As these young people have already taken the initial step to attend this mental health service, it is likely that they have already overcome this potential barrier. Greater differences may be noted if this measure was tested on a non-clinical sample or on those contemplating seeking help but not yet feeling comfortable or capable of doing so. Similarly, youth ratings of control were already high in the TAU group which potentially explains the lack of differences in this measure. It is important to note that some young people took the opportunity to make qualitative comments about the lack of focus by clinicians on the areas they had flagged as important in myAssessment; for example: “Well, I think we didn’t use it too much. I’d put stuff down in myAssessment and I’d go into my session and nothing was talked about that had anything to do with myAssessment”, and “It’s really useful if the clinician’s read through it and did bring up the things that you want to talk about. …but that’s [myAssessment] not even mentioned” [48]. Low clinician responsiveness has been an issue identified in earlier psychosocial screening related research, with responsiveness often reducing as the number or severity of issues increased [43, 49]. As such, although not identified as a significant concern in the current trial, it is important that any future implementation strategies include specific training to support clinicians to adequately address all issues raised by the young person.

The results also failed to support the hypothesis, and findings of previous research [35], that myAssessment will improve clinician rates of time efficiency and/or the ability to formulate a clear treatment plan. Some young people were quite slow to complete the application and this began to creep into the allocated session time. In these instances it is likely that clinicians, understandably, did not believe the application was a tool to improve time efficiency. Additionally, it is unlikely that clinicians had sufficient time to review the summary prior to seeing the young person. Consequently, future implementation strategies of the current or any alternative electronic psychosocial assessments must ensure that young people arrive early enough to provide adequate completion time, or are able to complete the assessment prior to the appointment.

Finally, the current research tested the effect of myAssessment on ratings of the therapeutic alliance. Clinicians had previously been concerned that incorporating technology so early in the therapeutic process would be detrimental to the therapeutic relationship [35], and with research consistently linking the therapeutic alliance to positive outcomes in therapy, this was a valid concern [50, 51]. However, the results of the current study show that their fear is unfounded. In both the TAU and Intervention phases, young people rated the Therapeutic alliance at levels considered ‘moderate’ [41]. In fact, although not statistically significant, there was a trend whereby ratings were higher when myAssessment had been used; the application was certainly not found to be detrimental to the important therapeutic relationship.

### Limitations and future research

This research was conducted as an initial pilot trial to determine future prospects of this type of technology. As the testing only occurred in a single service location it is not clear how widely these results may generalize. In particular, headspace is a relatively new youth friendly service willing to try innovative technologies and methods of service delivery to best meet the needs of young people [5, 6]. Consequently, the application may have been more readily accepted in this service setting. As it was not clear whether all clinicians strictly adhere to the verbal headspace Psychosocial Assessment for Youth, it could also not be determined how much of the increase in reporting was due to young people feeling more comfortable in the electronic format compared to being asked questions in a more systematic approach. As the sample was already attending headspace, it is also not clear whether the option of self-disclosing in this less threatening way may increase help-seeking in those who are currently apprehensive due to their limited ability to self-disclose. Another limitation concerns the collection of data sequentially without a concurrent control group. Although comparison of demographic data across both groups showed few differences, it is possible that differences in reported rates of substance use or risky behaviors in the Intervention phase could be due to changes in presenting issues over time. Further, due to an ethical requirement to protect the anonymity of the clinician participants, data could not be analyzed for clinician clustering effects. Consequently, it is unclear whether differences in verbal reporting were dependent on individual clinicians or their qualification backgrounds; although, as all clinicians were employed at the same service location with the same training and support, transfer of information between clinicians should be high and differences would likely be minimal.

Future research should continue to test the utility of this type of technology in more varied health settings and with those not currently seeking help. Future testing across services should include cluster analyses to determine whether differences are due to variances between clinicians or organizations, or entirely due to the mode of disclosure. In order to more clearly determine the reason for the increase in reported levels of behaviors it would also be useful to compare assessment technology against a carefully standardized structured verbal interview. Additionally, it would be beneficial to understand and develop the required implementation and training strategies to support the incorporation of new technologies into services that may be less willing to adopt new models of service delivery. Implementation strategies that address the clinical benefits of using technology for assessment, help clinicians understand how the additional information can support treatment planning, and ensure sufficient time for administration, may also improve future clinician acceptance and uptake of the technology.

## Conclusion

Electronic psychosocial assessments can be incorporated into standard face-to-face service delivery with widespread acceptability by young people and clinicians. Using technology to conduct psychosocial assessments will increase the likelihood of receiving accurate information on current and past behaviors and experiences without detrimentally affecting the therapeutic alliance. Young people are also more likely to believe there is greater time in session to talk about what they feel is most important. Receiving this added information so early in therapy is likely to improve service delivery and health outcomes.
